# Associations of internalized and anticipated HIV stigma with returning to work for persons living with HIV

**DOI:** 10.1371/journal.pone.0252783

**Published:** 2021-06-04

**Authors:** Joseph S. Lightner, Serena Rajabiun, Howard J. Cabral, Jessica Flaherty, Jamie Shank, Ronald Brooks

**Affiliations:** 1 School of Nursing and Health Studies, University of Missouri-Kansas City, Kansas City, Missouri, United States of America; 2 HIV Services, Kansas City Health Department, Kansas City, Missouri, United States of America; 3 Department of Public Health, Zuckerberg College of Health Sciences, University of Massachusetts, Lowell, Massachusetts, United States of America; 4 Department of Biostatistics, School of Public Health, Boston University, Boston, Massachusetts, United States of America; 5 School of Social Work, Boston University, Boston, Massachusetts, United States of America; 6 Center for HIV Identification, Prevention and Treatment Services, University of California Los Angeles, Los Angeles, California, United States of America; York University, CANADA

## Abstract

**Introduction:**

Employment is particularly beneficial for persons living with HIV (PLWH). However, PLWH experiencing internalized stigma or anticipating that they may experience stigma may be less likely to seek employment due to additional barriers associated with HIV. The purpose of this study was to understand the associations between internalized and anticipated stigma and employment barriers for PLWH.

**Methods:**

Participants (N = 712) from 12 sites across the United States were recruited and interviewed about barriers to employment, HIV stigma, and several other factors related to health. A series of unadjusted and adjusted linear regression models were conducted using cross-sectional data.

**Results:**

Adjusted models suggest that greater anticipated stigma was related to increased employment barriers (β = 0.12, p = 0.04). Mental and physical health functioning also positively predicted employment barriers (β = -0.18, p <0.001; β = -0.40, p <0.001, respectively).

**Discussion:**

Employment among PLWH has beneficial impacts on HIV-related health outcomes. This study suggests that anticipated stigma may limit and individual’s willingness to seek out employment, or may cause them to leave employment. Internalized stigma may not play as large of a role in employment as anticipated stigma for PLWH. HIV-related stigma reduction interventions focused on community-level and employers are essential to improve employment opportunities for PLWH.

## Introduction

About 1.2 million individuals are living with HIV in the United States [1]. Of those, 45–65% are unemployed and 10–20% are homeless [2]. In 2011, among persons living with HIV (PLWH), the majority were living at or below the federal poverty level, leaving this disadvantaged group with limited resources [3]. Employment opportunities for PLWH may help to address issues of poverty, homelessness, and limited resources in this population.

Due to effective medical treatments, PLWH are expected to live 43.1 years longer than individuals living with HIV just 20 years ago [4]. This increased life expectancy provides PLWH with an opportunity to retain or gain employment to live a life similar to those not infected with HIV and to help sustain normal activities.

Employment has been shown to be a major social determinant of health, particularly important for the financial and mental well-being of PLWH [5]. Results of a previous study suggest that those who are unemployed experience poorer psychological health in comparison to those working [6]. Loss of income associated with unemployment can negatively influence a person’s access to health care [7]. Unemployment has been linked to mental health disorders such as depression and suicidal ideation [8]. PLWH are no exception. In fact, greater quality of life has been associated with paid employment and having a higher level of income among PLWH [9]. Employment among PLWH may prevent disease progression by increasing one’s sense of belonging, access to health care, and medication adherence. Interventions to increase employment and income are needed for PLWH [10].

Research findings suggest that stigma and discrimination are significant barriers to employment for PLWH [11]. While 74% of unemployed PLWH were thinking of returning to work, 66% fear workplace discrimination as a potential barrier [12]. Stigma is particularly damaging to persons living with HIV (PLWH). PLWH who experience internalized, anticipated, and enacted stigma are more reticent to enter the workforce [13]. Among employers, previous research findings have associated fear of contagion and HIV-related stigma as factors in decisions to hire PLWH [11].

HIV stigma are negative attitudes, judgements, and discriminatory behaviors directed towards those at risk for or living with HIV [14]. Internalized HIV stigma is a negative self-perception of self-image due to HIV and has been be linked to poor physical, mental and cognitive health, and poor antiretroviral adherence [15]. The perception of HIV stigma within a community, such as an unfamiliar work environment, can deter PLWH from entering the workforce. An ecological momentary assessment study of HIV-positive gay and bisexual men revealed a positive correlation between experiences of internalized HIV stigma and negative affective experiences, such as depression, fatigue, anger, and emotion dysregulation [11]. Anticipated stigma are expectations of future repercussions from others due to HIV status including friends, family, and neighbors in the community. Anticipated stigma predicts healthcare behaviors and interpersonal outcomes [15].

A gap in the literatures exists for an individual’s perception of HIV stigma and employment barriers especially in populations that experience housing instability. The purpose of this study is to understand the associations among internalized and anticipated HIV stigma and employment barriers. We hypothesize that, consistent with existing literature, higher rates of stigma will be associated with more employment barriers.

## Materials and methods

The Heath Resources and Services Administration, HIV/AIDS Bureau, Special Projects of National Significance Initiative “Improving HIV Health Outcomes through the Coordination of Supportive Employment and Housing Services” funded 12 sites to deliver interventions focused on connecting PLWH to employment opportunities. All sites were located in urban areas with interventions based at outpatient HIV clinics or hospitals (4), city or county health departments (4) or AIDS Service Organizations (4). Common elements of the interventions included: i. employing navigators as part of the care team to support participants with obtaining necessary housing, employment and other social services, and ii. system level coordination with formal meetings between housing, employment and medical providers to share and exchange information and supported referrals [16].

Eligibility criteria included: 1) PLWH who is 18 years or older, 2) being homeless, unstably housed, imminent risk of losing housing or fleeing domestic violence, and 3) un- or under employed. Under employed was defined as not having enough paid work or not doing work that makes full use of their skills and abilities to meet their essential needs. In addition, participants had to have at least one of the following HIV health outcomes: 1) newly diagnosed with HIV in the past 12 months, 2) had been previously out of care for at least six months, or not virally suppressed at the time of enrollment. Participants were identified from clinic records and databases for out of care and virally suppressed candidates, from referrals from case managers and other internal team members and external partners.

### Data collection

Data collection was from May 2018-June 2019 with participants consenting to the study and completing a baseline interview administered by trained staff prior to being enrolled in the intervention. All data collection was conducted via in-person interviews by trained staff. Participants received a $25 gift card for interview completion and then were referred to intervention staff or introduced through a warm hand off to participate in the intervention. Study staff also gathered medical chart data to assess baseline viral load and last HIV primary medical visit for non-newly diagnosed clients. All client data were gathered using Research Electronic Data Capture (RED Cap, Version 8.6.4 Vanderbilt, Nashville, TN), a data management software program [17].

### Measures

Our main outcome was employment barriers gathered from a 13-item scale on perceived barriers to returning to work for people with HIV [12]. Barriers included fear of HIV discrimination and disclosure; inability to perform job duties due to poor health, lacking skills, or added stress; losing government benefits and/or health insurance; inability to get health insurance from their employer, and other aspects of employment. For each item, participants were asked yes/no if the item is a barrier to returning to work. The items were then summed and scored 0–13 with 0 being no barriers to high level of barriers.

Our independent variables were anticipated and internalized HIV stigma using a 15-item scale adapted from previously validated scales [13]. Internalized stigma was measured with six items, anticipated stigma included nine items all measured on a 5-point Likert scale with higher scores indicated greater stigma.

Additional covariates include socio-demographics, age, race/ethnicity, educational status, income, and sexual orientation. Current housing status was measured as literally homeless, unstably housed or imminent risk of losing housing. Current employment status was measured as working full/part time, underemployed, or unemployed. Unmet needs for 12 basic needs were collected that included food, clothing, housing, transportation and other needs. Social support was measured using five items on a 5-point Likert scale with higher scores indicating a greater need for social support in the past four weeks [18]. Food security for the past 6 months was measured with a 6-item scale with higher scores indicating higher food insecurity risk [19]. Addiction severity was measured using the World Health Organization’s ASSIST tool to assess lifetime substance use including tobacco, alcohol, and elicit substances [20]. For each substance used in an individual’s lifetime, additional information for the past 3 months is collected and an overall summary score is computed with a higher score indicating greater addiction severity [20]. Depression risk for the past week was measured using a 10-item short form on a 4-point Likert scale with higher scores above 10 indicating moderate to high risk for depression [21]. HIV medication adherence was measured by asking individual to rate their ability to take all HIV medications as prescribed in the last month on a scale from excellent to very poor. Other social and health risk factors included: mental health treatment and substance use treatment for the past 3 months; financial assistance, job training, legal and interpretation services in the last 6 months; lifetime history of incarceration; lifetime exposure to trauma [22]; current health insurance status (yes/no); and physical and mental health-related quality of life in the past 4 weeks [23]. All validated tools were used based on their recommended time frame. Viral load was collected from medical records in the past 6 months. Viral suppression was defined as having a HIV viral load less than 200 copies/ml.

### Data analysis

Of the 1083 individuals who participated in this study, 712 were seeking employment and asked about their perceptions of barriers to employment. Therefore, we only included participants who have data on employment barriers for this analysis. We conducted an inter-item reliability analysis on the 13-item barrier scale to ensure appropriate reliability. The hypothesis of this study was tested using two linear regression models. An unadjusted model was conducted with anticipated and internalized stigma as independent variables and employment barriers as the dependent variable. An adjusted model included site, employment status, participant age, household income, identified gender, sexual orientation, race, education, HIV medication adherence, viral suppression, years living with HIV, social support, unmet need, addiction severity, depression, incarceration, trauma, physical heath functioning, and mental health functioning as covariates. All scales were validated for the population ensuring the robustness of this study. Missing data were removed from analysis. All analyses were performed in SPSS, version 25 [24].

The study was approved by the Institutional Review Boards (IRB) at the University of Missouri-Kansas City (protocol: 2006108) and Boston University Charles River Campus (protocol: 4798E), as well as the 11 other participating sites (AIDS Foundation, Chicago; Avenue 360, Houston; Bexar Co Hospital System, San Antonio; City of Pasadena, California; City of Paterson, New Jersey; Family Health Centers, San Diego; Fenway Health, Boston; Gay Men’s Health Crisis, New York; Positive Impact Health Center, Atlanta; PRC, San Francisco; and Yale University, New Haven). Written documentation of consent was obtained by 11 sites. One site was granted a waiver of documentation of consent as the research was determined to be minimal risk.

## Results

Table 1 includes descriptive statistics, demographic characteristics, and other study variables. The sample of participants were, on average, 38.9 years old (SD 11.3 years) with annual household income of $7,806 (SD $11,856). The majority of the sample identified as male (79.0%), lesbian, gay, or homosexual (50.2%), and reported being racial or ethnic minorities (88.8%). Nearly half (48.8%) reported their highest level of education as high school or less.

**Table 1:** Demographic and other study variables (N = 712).

Variable		Mean or N	SD or %
Age (years)		38.9	11.4
Annual Household Income (USD)		7805.8	11856.4
Gender			
	Male	562	79.0%
	Female	114	16.1%
	Transgender or Other	35	4.9%
Sexual Orientation			
	Heterosexual or Straight	238	33.6%
	Lesbian, Gay, or Homosexual	356	50.2%
	Bisexual	87	12.3%
	Other	28	3.9%
Race			
	African American/Black	307	43.4%
	Hispanic	237	33.7%
	White	80	11.4%
	Other	83	11.7%
Education			
	Less than high school	138	19.4%
	High school	209	29.4%
	Some college/2-year degree/technical school	282	39.7%
	4-year degree or beyond	82	11.5%
Employment Status			
	Currently employed	0	0.0%
	Underemployed	141	19.8%
	Unemployed	570	80.1%
	Not currently seeking employment	1	0.1%
Housing Status			
	Literally homeless	296	41.7%
	Imminent risk of losing housing	78	11.0%
	Unstably housed/at risk of losing housing	319	44.9%
	Stably housed	17	2.4%
Viral Suppression*			
	Yes	219	67.4%
	No	106	32.6%
Years Living with HIV		9.4	8.3
Health Insurance			
	Yes	481	69.6%
	No	210	30.4%
Social Support		11.6	5.2
Unmet Needs		3.8	2.2
Addiction Severity Score		38.5	31.9
Depression Score		4.1	2.3
Incarceration history			
	Yes	212	29.9%
	No	497	70.1%
Total Trauma Score		4.1	2.3
Physical Health Functioning		44.3	12.5
Mental Health Functioning		35.3	6.9
HIV Stigma (scale 1low-5high)			
	Anticipated	2.3	0.9
	Internalized	2.5	1.1
Employment Barriers (summed scale 0–13)		5.3	3.1

*54.4% of viral suppression data are missing.

A majority of participants reported being unemployed (80.1%) or underemployed (19.8%) and literally homeless (41.7%) or unstably housed/at risk of losing housing (44.9%). The average reported scores of anticipated and internalized HIV stigma were 2.3 (SD 0.90) and 2.5 (SD 1.1), respectively. Most participants were virally suppressed (67.4%) and had health insurance (69.6%). Roughly, one-third of the sample reported being incarcerated at some point in their lives. Participants reported an average of 3.8 (SD 2.2) unmet needs out of a possible 13. Additionally, participants reported 5.3 (SD 3.1) barriers to employment out of a possible 13. Inter-item reliability for the 13-item barriers to employment scale showed appropriate reliability (alpha = 0.75) [25]. All other average values of covariates are presented in Table 1.

Results from the regression analyzes are presented in Table 2. First, we estimated and tested the unadjusted associations between anticipated and internalized stigma and employment barriers in Model 1. Anticipated stigma (β = 0.19, *p* = 0.001) and internalized stigma (β = 0.17, *p* = 0.002) were positively associated to employment barriers.

**Table 2:** Relationship between stigma and employment barriers.

**Model 1. Unadjusted Associations between Stigma and Employment Barriers**					
**Parameter**	**β**	**t**	***p***	**95% Confidence Interval**	
				**Lower Bound**	**Upper Bound**
Anticipated Stigma	0.19	3.26	0.001	0.25	1.02
Internalized Stigma	0.17	3.07	0.002	0.17	0.79
**Model 2. Adjusted Associations between Stigma and Employment Barriers**					
Anticipated Stigma	0.12	2.07	0.04	0.02	0.78
Internalized Stigma	0.07	1.15	0.25	-0.13	0.50

Note: N = 712; Model 1 R^2^ = .08, df = 2; Model 2 R^2^ = .20, df = 23

Second, we estimated and tested the associations between anticipated and internalized stigma and employment barriers while adjusting for covariates. The results are presented in Model 2. After adjusting for covariates, anticipated stigma was significantly and positively associated with employment barriers (β = 0.12, *p* = 0.04). After adjusting for covariates, internalized stigma was not related to employment barriers (β = 0.07, *p* = 0.25). Physical health functioning and mental health functioning also significantly predicted employment barriers (β = -0.40, *p* <0.001; β = -0.18, p <0.001, respectively) with higher physical and mental health functioning being associated with fewer employment barriers.

Model 1 and Model 2 significantly accounted for 8% and 20% of the total variation in employment barriers, respectively.

## Discussion

In the present study, we analyzed the associations between HIV-related stigma and employment barriers among PLWH. Anticipated stigma was related to employment barriers, such that higher levels of anticipated stigma were related to more barriers to employment adjusting for socio demographic and other risk factors. The study findings are consistent with existing literature [13]. The perception of being stigmatized because of HIV status can potentially deter PLWH from seeking or maintaining employment. This is significant as employment is associated with improved quality of life, less depression, and increased HIV medication adherence [13].

To our knowledge this is the first study that highlights the relationship of anticipated stigma and employment among people living with HIV. Previous studies have documented an association between internalized and anticipated stigma on adherence to HIV care visits and medications and physical health and well-being [26–28]. A recent review highlighted the role of employment as a social determinant affecting movement along the HIV care continuum for people living with HIV [29]. Our study suggests that for people living with HIV especially those from lower income racial ethnic minority communities, addressing the underlying consequences of stigma are essential to support not only physical and mental health but also economic well-being. Future studies are warranted to explore the mechanisms between stigma, employment and HIV health outcomes.

Previous studies suggest that there is strong evidence employment is therapeutic and combats negative feelings associated with unemployment [30]. Throughout the life span, employment among PLWH has been shown to be associated with better disease-specific health factors and higher neurocognitive functioning [31–33]. Since employment may be beneficial for improvement of quality of life and well-being, elimination of HIV stigma and employment barriers among PLWH is essential. Future research is needed to understand the complex nature of employment and health for PLWH to determine potential spuriousness of age, health status, and other factors.

Currently, health disparities are universally recognized, with increased rates of mortality and disease burden disproportionately falling upon racial/ethnic minorities and the poor [33]. Among these vulnerable individuals are PLWH and those at risk for contracting HIV. HIV infection is most prevalent among persons with limited resources and does not have the same impact among those within the higher income level [30]. Our sample of PLWH are from racial/ethnic minority communities experiencing unstable housing and are in need of employment. Stigma and specifically anticipated stigma not only affects access and use of health care but limits employment opportunities.

There are many strengths to this study. First, we recruited a large, national sample of PLWH who were experiencing housing instability, low-income, and unemployment or under-employment. Our findings may be generalized to other low-income, unemployed PLWH in the United States experiencing housing instability.

However, limitations of this study include the self-report nature of barriers to employment and perceptions of stigma. This study is cross-sectional and, as such, we cannot determine causation. A significant portion of viral suppression data are missing due to the cross-sectional nature of this study. We did not impute any missing data in this analysis. The majority of the participants in this study are men, and thus the results may not be representative of women living with HIV and barriers to employment. Additionally, the results may not represent other higher income, higher education populations with access to resources.

Further research should attempt to understand the causal relationship between HIV stigma and barriers to employment. Additionally, an intersectional investigation of multiple form of stigma that PLWH experience is warranted. Developing interventions aimed at reducing anticipated HIV stigma may provide opportunities and stability in employment and improved quality of life. We also suggest exploring employer perceptions of stigma, as well as openness and support for employees living with HIV. Perhaps, by focusing on both community-level and employer interventions, practitioners may reduce the severity and impact of anticipated stigma on employment.

## Supporting information

S1 Data Data release. (XLSX)

S1 Table Supplemental table. (DOCX)
