# Supplementary material for: Associations of internalized and anticipated HIV stigma with returning to work for persons living with HIV
Source: PLoS One. 2021 Jun 4;16(6):e0252783. doi: 10.1371/journal.pone.0252783 (PMC8177530; doi:10.1371/journal.pone.0252783)
Supplement: S1 Table — (DOCX) [file pone.0252783.s002.docx]

S1 Table. Supplemental Table.

| **Model 1. Unadjusted Associations between Stigma and Employment Barriers** | | | | | |
| --- | --- | --- | --- | --- | --- |
| **Parameter** | **β** | **t** | ***p*** | **95% Confidence Interval** | |
|  |  |  |  | **Lower Bound** | **Upper Bound** |
| Anticipated Stigma | 0.19 | 3.26 | 0.001 | 0.25 | 1.02 |
| Internalized Stigma | 0.17 | 3.07 | 0.002 | 0.17 | 0.79 |
|  | | | | | |
| **Model 2. Adjusted Associations between Stigma and Employment Barriers** | | | | | |
| Anticipated Stigma | 0.12 | 2.07 | 0.04 | 0.02 | 0.78 |
| Internalized Stigma | 0.07 | 1.15 | 0.25 | -0.13 | 0.50 |
| Site | 0.02 | 0.30 | 0.77 | -0.08 | 0.11 |
| Employment Status | -0.05 | -0.88 | 0.38 | -0.67 | 0.26 |
| Housing Status | 0.07 | 1.42 | 0.16 | -0.09 | 0.57 |
| Age | 0.05 | 0.85 | 0.40 | -0.34 | 0.86 |
| Income | 0.03 | 0.55 | 0.59 | -0.29 | 0.52 |
| Gender | 0.02 | 0.39 | 0.70 | -0.47 | 0.70 |
| Sexual Orientation | 0.04 | 1.03 | 0.30 | -0.10 | 0.33 |
| Race | 0.03 | 0.53 | 0.60 | -0.27 | 0.48 |
| Education | -0.00 | -0.04 | 0.97 | -0.37 | 0.35 |
| Insurance | 0.02 | 0.45 | 0.66 | -0.59 | 0.93 |
| HIV Medication Adherence | -0.03 | -0.61 | 0.54 | -0.27 | -0.14 |
| Viral Suppression | 0.01 | 0.20 | 0.84 | -0.63 | 0.78 |
| Years Living with HIV | 0.02 | 0.31 | 0.75 | -0.03 | 0.05 |
| Social Support | -0.07 | -1.30 | 0.19 | -0.11 | 0.02 |
| Unmet Needs | 0.05 | 0.88 | 0.38 | -0.08 | 0.22 |
| Addiction Severity Score | -0.00 | -0.07 | 0.94 | -0.01 | 0.01 |
| Depression Score | 0.04 | 0.58 | 0.56 | -0.04 | 0.07 |
| Incarcerated history | -0.01 | -0.16 | 0.87 | -0.81 | 0.69 |
| Total Trauma Score | -0.02 | -0.37 | 0.71 | -0.18 | -.12 |
| Physical Health Functioning | -0.40 | -6.15 | <0.001 | -0.12 | -0.06 |
| Mental Health Functioning | -0.18 | -3.36 | 0.001 | -0.13 | -0.03 |
| Note: N = 712; Model 1 R^2^ = .08, df = 2; Model 2 R^2^ = .20, df = 23 | | | | | |
